# Epigenetics: New Questions on the Response to Hypoxia

**DOI:** 10.3390/ijms12074705

**Published:** 2011-07-21

**Authors:** Joel I. Perez-Perri, Julieta M. Acevedo, Pablo Wappner

**Affiliations:** 1 Instituto Leloir, Patricias Argentinas 435, Buenos Aires C1405BWE, Argentina; E-Mails: jpperri@leloir.org.ar (J.I.P.-P.); jacevedo@leloir.org.ar (J.M.A.); 2 Consejo Nacional de Investigaciones Científicas y Técnicas, Buenos Aires C1033AAJ, Argentina; 3 Facultad de Ciencias Exactas y Naturales (FCEyN), Universidad de Buenos Aires, Buenos Aires C1428EGA, Argentina

**Keywords:** HIF, oxygen, stress, chromatin, histone, jumonji

## Abstract

Reduction in oxygen levels below normal concentrations plays important roles in different normal and pathological conditions, such as development, tumorigenesis, chronic kidney disease and stroke. Organisms exposed to hypoxia trigger changes at both cellular and systemic levels to recover oxygen homeostasis. Most of these processes are mediated by Hypoxia Inducible Factors, HIFs, a family of transcription factors that directly induce the expression of several hundred genes in mammalian cells. Although different aspects of HIF regulation are well known, it is still unclear by which precise mechanism HIFs activate transcription of their target genes. Concomitantly, hypoxia provokes a dramatic decrease of general transcription that seems to rely in part on epigenetic changes through a poorly understood mechanism. In this review we discuss the current knowledge on chromatin changes involved in HIF dependent gene activation, as well as on other epigenetic changes, not necessarily linked to HIF that take place under hypoxic conditions.

## 1. Introduction

Metazoans organisms utilize molecular oxygen during cellular respiration to produce sufficient amounts of ATP, as continuous and proper oxygen supply is an absolute requirement for survival. In response to hypoxia, defined as the reduction of oxygen levels below normal values, complex mechanisms are triggered to allow adaptation to this environmental condition. These adaptive responses comprise changes at the systemic, tissue and cellular levels that tend to reduce oxygen consumption, and improve oxygen supply. Cardinal adaptations to hypoxia include angiogenesis, erythropoiesis, a switch from oxidative to glycolytic metabolism, and a partial shut-down of major energy consuming cellular processes [1–3].

Remarkably, hypoxic conditions take place during normal physiological processes, being a necessary stimulus for embryonic development and stem cell maintenance [4–7]. During different human pathological conditions, hypoxia also plays a major role. The pathologies include cancer, stroke, myocardium infarction and chronic kidney disease being the most relevant ones [3,8–11].

The cellular response to hypoxia is largely dependent on changes in gene expression, which are mainly commanded by a unique family of transcription factors named HIFs, for Hypoxia Inducible Factors [12–13]. HIFs are heterodimeric transcription factors composed of a constitutively expressed HIF-1β subunit and one of three oxygen-sensitive alpha subunits (HIF-1α, HIF-2α or HIF-3α) [14–15]. Under normal oxygen levels (normoxia), HIF-α subunits are hydroxylated in two key prolyl residues by specific prolyl-hydroxylases named PHD1, PHD2 and PHD3 [16–18]. These enzymes utilize molecular oxygen and 2-oxoglutarate as co-substrates for the reaction [18]. Hydroxylated HIF-αs are recognized by the von Hippel-Lindau protein (VHL), the substrate recognition subunit of an E3 ubiquitin ligase complex; HIF-αs are then ubiquitinated and degraded at the 26S proteasome [19–21]. Under hypoxia, when oxygen is limiting, PHD activity is reduced, HIF-α subunits escape hydroxylation and proteolysis, leading to heterodimerization with the β-subunit and induction of hundreds of target genes through the binding of specific DNA sequences termed HREs (for Hypoxia Response Elements) [14]. Whereas HIF target genes are upregulated under oxygen deprivation, general transcription in the cell is largely inhibited [22].

Eukaryotic DNA is complexed with different histone and non-histone proteins to form the chromatin [23–24]. Histones play an essential role in DNA compaction as well as in the regulation of all DNA-related processes, including transcription, replication and DNA repair. The structural and functional unit of the chromatin is the nucleosome, which consists of an octamer of two molecules of each of the histones H2A, H2B, H3 and H4, around which 147 base pairs of DNA are wrapped [23–24]. Nucleosomes are connected by linker DNA which is associated with linker histones, usually H1 and H5 [24]. Nucleosomes together with the linker DNA are progressively folded and compacted into structures of higher-order [25–27]. Highly compacted chromatin has an inhibitory effect on transcription, since it limits the accessibility of the transcriptional machinery to the DNA [26]. All nucleosomal histones have a globular domain that forms the nuclesome core, and an N-terminal tail that protrudes away from the DNA [23]. Histone tails are subjected to multiple posttranslational modifications, including acetylation, methylation, phosphorylation and ubiquitination [28–29]. These histone marks have effects on both chromatin compaction and recruitment of different proteins that regulate transcription [30–31].

Besides the posttranslational modifications of the histone tails, chromatin can also be modified by various ATP-dependent chromatin remodeling complexes, which change the accessibility of the transcription machinery to particular loci, by promoting nucleosome sliding, nucleosome exchange or DNA exposure [32–34].

Alteration of the gene expression profile is a common cellular response to different types of stress. Changes in gene expression under stress are intimately associated with alterations in chromatin structure, mediated by histone modifying and chromatin remodeling complexes [35]. In this review we will discuss the chromatin alterations that take place under hypoxia and the mechanisms by which they regulate gene expression to restore homeostasis under this condition.

## 2. Role of HIF Co-Factors in the Response to Hypoxia

In order for transcription to take place, an initiation complex conformed by the RNA Polymerase II and general transcription factors must overcome the physical barrier posed by the chromatin. It has been demonstrated that HIF recruits co-activators that modify the chromatin structure thereby facilitating access of the transcriptional machinery to the DNA. These so called HIF co-activators include the histone acetyltransferases (HAT) p300 and CREB-binding protein (CBP) [36–40], some histone deacetylases (HDACs) [41–42] and the chromatin remodeling complex SWI/SNF [43].

### 2.1. Role of Histone Acetyltransferases and Histone Deacetylases in the Response to Hypoxia

Histone acetylation, a process strongly associated with transcriptional activation, takes place on lysine residues localized in histone N-terminal tails [44]. Histone acetylation induces transcription by relaxing high-order structures of the chromatin. In addition, acetylated histones create signals for the binding of bromodomain-containing proteins, which frequently present intrinsic HAT activity or are members of chromatin remodeling complexes, thereby contributing to transcription induction [30,44–46].

p300 and CBP are two paralogues with strong HAT activity, highly conserved in most multicellular organisms [47]. In addition to promoting histone acetylation, p300 and CBP function as physical bridges between several transcription factors and basal transcription machinery elements, such as the TATA binding protein and TFIIB, enhancing transcriptional activation [47–49]. p300 and CBP have strong co-activator effect on HIF dependent transcriptional induction [36–40].

Both HIF-1α and HIF-2α have been shown to interact with p300/CBP through two distinct transactivation domains termed C-TAD [50–51] and N-TAD [52]. HIF C-TAD, placed next to the HIF carboxy-terminal end, interacts with a p300/CBP domain named CH1. The more centrally localized HIF N-TAD interacts with a different p300/CBP protein domain called CH3 [50–52].

**Figure 1:**
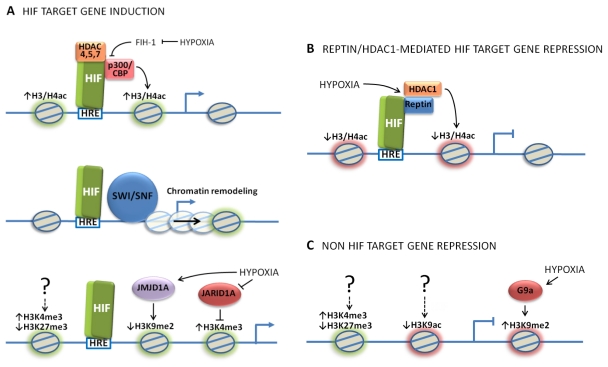
Epigenetic regulation of transcription in response to hypoxia. Schematic representation of the different epigenetic mechanisms involved in the expression of different genes in response to hypoxia. (a) HIF recruits co-activators that enhance gene expression. Upper panel: p300/CBP histone acetyltransferases interact with HIF and acetylate histones in HIF target promoters. HIF-p300/CBP interaction is induced in hypoxia through FIH-1 inhibition. HDAC4, HDAC5 or HDAC7 form a multiprotein complex with HIF-p300 increasing HIF transcriptional activity. HDAC4 and HDAC5 exert their effects by promoting association between HIF and p300. Middle panel: The SWI/SNF complex alters the chromatin structure in some HIF target promoters or enhancers, thereby favoring their expression. Lower panel: Hypoxia promotes changes in the histone methylation status at promoters of hypoxia-inducible genes: Oxygen deprivation activates JMJD1A and inhibits JARID1A histones demethylases, which provoke respectively a decrease in H3K9me2 and an increase in H3K4me2 levels at their target promoters, thus enhancing gene expression. In addition, hypoxia increases H3K4me3 and H3K27me3 levels in some HIF target promoters through an unknown mechanism; (b) Under hypoxia, the interaction between Reptin and HIF1-α is enhanced, leading to recruitment of HDAC1 to some HIF target genes, negatively regulating their transcription; (c) The histone methylation and acetylation status changes in promoters of hypoxia-repressed genes. Hypoxia provokes an increase in H3K9me2 levels as a result of G9a up-regulation. Increased H3K4me3 levels and decreased H3K27me3 and H3K9ac levels have also been observed. Epigenetic events typically associated with transcriptional repression are highlighted in red and those usually associated with transcriptional activation are highlighted in green. Abbreviations: HDAC, Histone Deacetylase; H3, histone H3; me2/3, di/tri-methylated; ac, acetylated.

Interaction between p300/CBP CH1 and HIF C-TAD is regulated by oxygen, mainly through the hydroxylation of a single asparagine residue within the HIF C-TAD [53]. This hydroxylation, catalyzed by a hydroxylase termed Factor Inhibiting HIF (FIH-1) [54–55], prevents the interaction between HIF-α and p300/CBP. Like Prolyl-4-hydroxylases (PHDs), FIH-1 also requires molecular oxygen for catalysis. Under hypoxia, when oxygen availability is limiting, asparagine hydroxylation is inhibited, and the interaction between HIF and p300/CBP is restored, thereby allowing HIF dependent transcription in hypoxia (Figure 1A, upper panel). Interaction between the CH3 domain of p300/CBP and HIF-1α N-TAD does not seem to be oxygen-sensitive and it is significantly weaker than the CH1-C-TAD interaction [52,56].

In addition to FIH-1 dependent regulation, several other factors and pathways have been shown to modulate the interaction between p300/CBP and HIF-α subunits. These additional regulatory factors include positive regulators, such as casein kinase II and mitogen-activated protein kinase (MAPK), as well as negative regulators, including CITED2 andCITED4 [3,35,57]. Sirtuins are a family of nicotinamide adenine dinucleotide (NAD+)-dependent deacetylases that sense the redox state of the cell. Sirtuin1 (SIRT1) is an interesting modulator of HIF activity that negatively regulates HIF-1α [58] and positively regulates HIF-2α [59]. SIRT1 reduces HIF-1α activity by abrogating interaction between p300/CBP and HIF-1α [58]. Whether p300/CBP plays a role on SIRT1-mediated positive regulation of HIF-2α is unknown. Although p300/CBP have been shown to regulate expression of various HIF dependent reporters and several HIF target genes [36,40,60], as well as to display histone acetylation activity over a few HIF target promoters [43,61–62] (Table 1), a general requirement of these factors in HIF dependent transcription has not been demonstrated. Furthermore, work carried out by Kasper and colleagues [63] suggests instead, that the role of p300/CBP in the HIF-dependent cellular response to hypoxia may not be as general as predicted from studies carried out *in vitro*. These authors demonstrated that simultaneous deletion of the CH1 domain of both p300 and CBP provoked very diverse effects on the induction of 40 different HIF target genes. This implies that the requirement of this domain is gene-specific, being essential for some genes and dispensable for others. In agreement with this, a recent study has shown that individual knock down of p300 or CBP does not reduce hypoxic induction of the HIF target genes *LDH-A* and *PGK*, suggesting that p300 and CBP are either redundant or dispensable in the expression of these and possibly other HIF targets [62].

Why does deletion of the CH1 domain of the two HATs provoke only a moderate effect on the expression of certain HIF target genes? In principle, one explanation could be that p300/CBP are recruited to specific promoters through their intact CH3 domains. Nevertheless, this is apparently not the case, since CH1 deletion severely reduces the recruitment of p300/CBP to HIF target promoters, without significantly affecting gene expression. Thus, it seems likely that p300/CBP are dispensable for HIF dependent induction of a subset of HIF target genes [63].

Histone acetylation is controlled by the opposing activities of HATs and HDACs [69]. In mammals 18 different HDACs have been identified so far [70–73]. It is well known that HDAC substrates also include non-histone proteins, including several transcription factors and co-factors [74].

By targeting histones or non-histone proteins, HDACs generally have a negative effect on transcription [74], but interestingly, they can regulate HIF dependent transcription both positively or negatively. It was shown that under hypoxia, a HIF target gene named *RECK* is downregulated through a mechanism that involves recruitment of HDAC1 to its promoter [75]. It was shown in addition, that in hypoxia the chromatin-remodeling factor Reptin binds simultaneously to HIF-1α and HDAC1, recruiting in this way a repressive complex containing HDAC1 to a subset of HIF target genes [76] (Figure 1B). On the other hand, recruitment of HDAC4, HDAC5 and HDAC7 to HIF target promoters since has a positive role in HIF dependent transcription [41–42]. Consistent with this, histone deacetylase inhibitors, that in most cases promote gene expression, in the context of several HIF target promoters provoke inhibition of transcription [63,77] and of HIF-dependent angiogenesis [74,78–80]. The mechanism involved in HDAC-dependent gene activation is not well defined as yet. However, it is becoming increasingly clear that for HIF C-TAD-p300/CBP interaction to take place, a deacetylation reaction is required [74]. Available evidence suggests that p300/CBP and not HIF are targets of such deacetylation [77]. In line with this notion, HIF, p300 and HDAC4, HDAC5 or HDAC7 have been reported to form a multiprotein complex [41–42]. It was also shown that HDAC4 and HDAC5 promote association between HIF-1α and p300, thereby enhancing expression of HIF target genes [42] (Figure 1A, upper panel).

### 2.2. Role of Chromatin Remodeling in the Response to Hypoxia

Relatively little is known about the role of chromatin-remodeling complexes in the transcriptional response to hypoxia. To date, there are only two studies covering this issue, and both have focused in a unique complex termed SWI/SNF [43,81].

SWI/SNF utilizes energy derived from ATP hydrolysis to disrupt interactions between DNA and histones thereby changing chromatin structure, generally facilitating the access of DNA-binding proteins to their target sequences [82].

It has been demonstrated that components of the SWI/SNF complex are recruited to the *EPO* enhancer (a HIF target), and that this recruitment is required for full *EPO* induction under hypoxia [43] (Figure 1A, middle panel). It was shown in addition, that in hypoxia the SWI/SNF complex is recruited to the promoter of the HIF-1α gene and t hat t his is required for expression o f HIF-1α mRNA. Thus, this study concluded that modulation of SWI/SNF levels can account for deep changes in HIF-dependent responses to hypoxia [81]. We have recently carried out a genome-wide RNAi screen in *Drosophila* cells aimed to detect novel HIF activators. Several components of the SWI/SNF complex (called Brahma complex in *Drosophila*) have emerged as hits of the screen, suggesting that the requirement of the SWI/SNF complex in the transcriptional response to hypoxia is a conserved feature in animal evolution [83].

## 3. Role of Jumonji-Domain Containing Histone Demethylases in the Response to Hypoxia

Histones can be methylated in specific lysine (K) or arginine (R) residues, which can appear as mono-, di-, or tri-methylated forms. Methylated histone residues induce alterations in compaction of the chromatin, and also provide binding sites for different proteins that regulate gene expression, resulting in the inhibition or enhancement of transcription [84–87]. Although some exceptions have been reported [30,88], in general, methylation at lysines 4, 36 or 79 of H3 are hallmarks of chromatin active regions, whereas methylation of lysines 9 and 27 of H3, as well as of H4 lysine 20 are associated with transcriptional repression and heterochromatin formation [29,86]. Work carried out over the last ten years has shown that histone methylation is a tightly regulated process, dependent on the activity of both histone methyltransferases and histone demethylases (HDMs) [29,86].

The Jumonji C (JmjC)-domain containing histone demethylases (JHDM) constitute the largest family of lysine demethylases. JHDM family members utilize molecular oxygen (O_2_), Fe(II) and 2-oxoglutarate to remove methyl groups from specific histone residues through an hydroxylation reaction. Several lines of evidence have linked JHMD function to hypoxia [64,89–90].

As JHDMs utilize O_2_ as a co-substrate in the demethylation reaction, one could anticipate that their enzymatic activity is compromised under hypoxia. In agreement with this, it was demonstrated that the activity of over-expressed JMJD1A and JMJD2B was diminished, although not completely abrogated, when cells were exposed to 0.2% O_2_ [91]. Interestingly, most members of the JDHM family have been reported to be transcriptionally induced under hypoxic conditions [64,89,91–93]. It was therefore proposed that JHDM induction under hypoxia tends to restore histone methylation homeostasis, in such a way that when oxygen availability is limiting, by increasing the expression of these enzymes, reduction of enzymatic activity due to oxygen scarcity is totally or partially compensated [89]. In agreement with this model, 17 out of the 22 JHDM family members are induced under hypoxic conditions [89], and at least 4 of them, JMJD1A, JMJD2B, JMJD2C and JARID1B, are HIF direct targets, as evidenced by chromatin immunoprecipitation (ChIP) assays [64,89,91–93].

Results reported by Xia *et al*. [89] support a role of a JHDM named JARID1B in histone methylation homeostasis: They have shown that under hypoxia, genome-wide levels of tri-methyl lysine 4 of histone H3 (H3K4me3) are increased, and that this increase is enhanced if cells are additionally depleted from HIF-β. As the effect on histone methylation elicited by the depletion of HIF can be over-came by JARID1B overexpression, it was proposed that JARID1B HIF-dependent induction is necessary to prevent H3K4 hypermethylation under hypoxia. Considering that some JHDMs, including JMJD1A and JMJD2B, are induced by hypoxia to a higher extent than other members of the family [89,91–92], and taking into account that JHDMs display residue specificity, hypermethylation of some histone lysines under hypoxia could be specially prevented.

It has been proposed that in addition to a possible role of the JHDMs in global histone methylation homeostasis under hypoxia [89], some family members might play a direct role in specific gene expression [93]. In fact, Krieg and colleagues [64] have recently demonstrated that JMJD1A is necessary for full hypoxic induction of 53 genes, including several HIF targets such as *ADM*, *EDN1*, *SERPINE1*, *PLAUR* and *HMOX1*. Moreover, they have shown that JMJD1A contributes to hypoxia-dependent gene expression by demethylating H3K9me2 in certain hypoxia responsive promoters, such as *ADM* and *GDF15* [64] (Figure 1A, lower panel). Notably, JMJD1A recruitment reduces, although it does not completely prevent, hypoxic induction of H3K9me2 on selected promoters [64].

It was reported that hypoxia induces global histone methylation possibly through partial inhibition of JHDMs [22,65,67], while it reduces histone methylation of certain hypoxia-responsive promoters [22]. Tausendschön *et al*. have shown that in macrophages, hypoxia provokes an increase in H3K9 di- and tri-methylation in promoter regions of several hypoxia-repressed genes, while hypoxia has no effect on these modifications in the promoter region of the hypoxia-inducible gene *ADM* [68].Gene-specific recruitment of JMJD1A (and probably other JHDMs) can explain this dual effect. It remains to be determined the molecular mechanisms that target JMJD1A to specific promoters.

Interestingly, JMJD1A is a HIF target gene itself. Krieg and colleagues [64] suggested that regulation of JMJD1A by HIF may represent a feed-forward mechanism for favoring HIF-dependent gene expression. They proposed that JMJD1A maintains an active epigenetic pattern in target promoters, thereby minimizing the energy required to support expression.

The specific regulation of just a subset but not all hypoxia-inducible genes by JMJD1A suggests that additional promoter-specific mechanisms account for differential gene induction. It is tempting to speculate that other JHDMs are also involved in the activation of specific hypoxia responsive genes. Further research is required to determine if this is indeed the case.

Although progress has been made in characterizing JHDM induction under hypoxia, detailed studies on the biological roles of this family of enzymes in the response to hypoxia needs to be carried out. RNAi silencing experiments targeting individual or multiple JHDMs, and posterior analysis of global as well as promoter-specific histone methylation will help to clarify the role of these enzymes in hypoxia-dependent regulation of transcription.

## 4. Gene-Specific Histone Modifications Induced under Hypoxia

Johnson *et al*. [22] analyzed hypoxia-induced epigenetic changes in the promoter regions of genes activated or repressed under oxygen deprivation. Hypoxic induction of the HIF target genes *VEGF* and *EGR1* correlates with a marked increase in H3K9ac and H3K4me3 levels and a decrease in H3K27me3 levels in their promoters, three events usually associated with transcriptional active loci (Figure 1A, lower panel). The same or similar epigenetic changes have also been observed at the promoters of the hypoxia-inducible genes *EPO*, *HMOX1* and *DAF* [43,62,65] (Table 1). Hypoxic induction of H3K4me3 seems to rely on the inhibition of the JARID1A demethylase [65].

In contrast, promoters of several hypoxia-repressed genes show decreased levels of H3K9ac [22,66] and/or increased levels of H3K9me2 [22,66–68] under hypoxia, events typically associated with transcriptional repression (Table 1). Available evidence suggests that H3K9me2 increase is supported in part by the hypoxic-dependent induction of the G9a methyltransferase [67] (Figure 1C).

Of interest, hypoxia also increased H3K9me2 levels at the promoters of some genes induced in hypoxia [64] (Table 1), as well as it provokes modifications typically associated with transcriptional activation (increased H3K4me3 and decreased H3K27me3 levels) at the promoters of some hypoxiarepressed genes [22] (Figure 1C and Table 1). These observations are not paradoxical, since patterns of histone modifications can be differentially interpreted by transcriptional factors and co-factors, depending on the epigenetic and cellular contexts [88]. Then, a so called “positive” histone mark can have repressive effects in particular physiologic situations, or *vice versa*, a typical “negative” modification can favor transcription in a certain physiologic context. Further research is required to assess the biological outcome of hypoxia-induced epigenetic changes.

## 5. Global Histone Modifications Induced under Hypoxia

In addition to promoter-specific chromatin alterations, cells exposed to hypoxia show global epigenetic changes [22,65–68,89,94–95]. Several groups have shown that the global acetylation and methylation profiles of H3 and H4 [22,67,94] and also methylation of the DNA (recently reviewed in [96]) are oxygen sensitive. Remarkably, among the hypoxia-induced global epigenetic alterations, histone posttranslational modifications usually associated to either transcriptional repression or activation have been reported [22,65–68,89,94–95] (Table 2).

It has been shown that hypoxia provokes general reduction of gene transcription [22]. However, it remains to be determined to what extent global epigenetic alterations account for this global gene downregulation in situations of oxygen shortage. Johnson and colleagues [22] have determined that HePa 1–6 cells incubated in strong hypoxia (0.2% O_2_ for 48 h) reduce their overall rate of transcription to about 50% of their usual normoxic levels. This sharp reduction in mRNA synthesis correlates with several histone modifications, that are not only associated with transcriptional repression, but also with transcriptional induction. Again, as a particular histone mark can promote different, even opposite biological effects, it cannot be ruled out that these “positive” histone marks globally induced under oxygen deprivation might have negative effects in the particular physiological and epigenetic contexts that take place under this condition.

An increase in di- and tri-methylation of H3K9, two typically repressive marks, are among the best characterized global modifications induced under hypoxia [22,66–68,94,97] (Table 2). Chen *et al*. [67] have demonstrated that the hypoxia-dependent increase in H3K9me2 stems from both increased methylation and decreased demethylation of H3K9. These authors have shown that at 0.2% O_2_ the activity of the methyltransferase G9a increases, supporting in part the accumulation of methylated species. In addition, they have shown that demethylation of H3K9me2 is reduced under hypoxia, although the identity of the particular demethylases whose activity diminishes is unknown. As has been mentioned above, JDHM activity is partially inhibited at 0.2% O_2_, so one possibility is that reduction of H3K9me2 demethylation is a consequence of partial loss of activity of one or more JHDM.

Remarkably, deacetylation of H3K9, another chromatin modification associated with transcriptional repression, globally increases under oxygen deprivation conditions [22,66–67,89,94]. Thus, taken together, these observations suggest that in hypoxia H3K9 acetyl groups are globally replaced by di- or tri- methyl groups establishing a mechanism that might account for the overall transcriptional repression seen in response to oxygen deprivation (Table 2).

In hypoxia, global induction of H3K4 di- or tri- methylation, both of them modifications usually associated with transcriptional activation, have also been well documented [22,66–68,89,94] (Table 2). Experiments carried out by Zhou *et al*. [65] suggest that hypoxia provokes an increase in H3K4me3 by inhibiting JARID1A demethylase activity, rather than by inducing methylation.

In summary, oxygen deprivation induces a widespread combination of histone modifications that are usually associated with either transcriptional repression or transcriptional activation. The field is now beginning to define more precisely these modifications, although more research is required to fully understand their biological roles, as well as to identify the enzymes and signaling pathways involved. It will be highly relevant to assess the impact of preventing the global epigenetic changes that occur under hypoxia, over general transcriptional repression and specific gene induction.

## 6. Concluding Remarks

In order to activate gene transcription, HIF recruits a range of gene-specific co-factors that acetylate/ormethylate histones, or change chromatin structure. It would be very interesting to define the mechanisms that recruit co-factors to specific hypoxia-inducible genes. On the other hand, the hypoxic stimulus induces a general repression of transcription, presumably supported by global chromatin changes. Therefore, it seems that epigenetic mechanisms play a dual role in gene regulation under hypoxia, controlling HIF target gene induction and the downregulation of general transcription.

## Figures and Tables

**Table 1:** Gene-specific histone modifications induced under hypoxia. Histone modifications induced at promoter regions of hypoxia-responsive genes. Up and down arrows indicate hypoxia-dependent increase or decrease of each of the modifications. Events typically associated with transcriptional repression are highlighted in red and those associated with transcriptional activation are in green. Abbreviations: me1/2/3, mono-/di-/trimethylation; ac, acetylation.

Gene	Event	References
Hypoxia-induced genes:		
*VEGF A*	↑ H3ac ↑ H3K4me3 ↓ H3K27me3	[22,61]
*EPO*	↑ H3ac ↑ H4ac	[43,62]
*EGR1*	↑ H3ac ↑ H3K4me3 ↓ H3K9/27me2 ↓ H3K27me3	[22]
*ADM GDF15*	↑ H3K9me2	[64]
*HMOX1 DAF*	↑ H3K4me3	[65]
*SP-A*	↓ H3ac ↑ H3K9me2	[66]
*AFP Albumin*	↓ H3ac ↑ H3K9/27me2	[22]
	↑ H3K4me3 ↓ H3K27me3	
*Mlh1 Dhfr*	↑ H3K9me2	[67]
*Ccl2* *Ccr1* *Ccr5*	↑ H3K9me2 ↑ H3K9me3	[68]

**Table 2:** Global histone modifications induced under hypoxia. Oxygen deprivation provokes global epigenetic changes usually associated with either transcriptional repression (red) or activation (green). Up and down arrows indicate that the corresponding modification is provoked or prevented by hypoxia. Abbreviations: me1/2/3, mono-/di-/trimethylation; ac, acetylation.

Histone	Residue	Modification	Event	References
**H3**	K4	me1	↑	[22]
		me2	↑	[22,89]
		me3	↑	[22,65,89]
	K9	ac	↓	[22,26,66–67]
		me1	↓	[67,94]
		me2	↑	[22,66–68,89,94]
		me3	↑	[67–68,94]
	K14	ac	↑	[22]
	K27	me3	↑	[22]
	K36	me3	↑	[68,89]
	K79	me2	↑	[22]
**H4**	R3	me2	↑	[22]
	K5,8,12,16	ac	↓	[22,94]
